# Supplementary material for: A Likelihood Approach for Real-Time Calibration of Stochastic Compartmental Epidemic Models
Source: PLoS Comput Biol. 2017 Jan 17;13(1):e1005257. doi: 10.1371/journal.pcbi.1005257 (PMC5240920; doi:10.1371/journal.pcbi.1005257)
Supplement: S1 File — (TAR.GZ) [file pcbi.1005257.s014.tar.gz › HSPH_Online-SI-Revision/PF08/n100-mild/n100-mild_PF2_table0.pdf]

1	newly 3 weeks spec	2866.	6567.86	{6249., 6825.}	129.165	0.359895	newly 3 weeks cumul	9776.	18242.8	{17217., 19002.}	86.6076	0.270647	newly 5 weeks spec	1628.	6321.8	{6148., 6522.}	288.317	0.58906	newly 5 weeks cumul	13712.	31161.8	{29785., 32327.}	127.259	0.356316	newly 52 weeks cumul 16805. 67497.7 {66165., 68626.} 301.653 0.603803
2	newly 3 weeks spec	3141.	6028.65	{5633., 6427.}	91.9341	0.282688	newly 3 weeks cumul	11118.	17657.8	{16720., 18824.}	58.8215	0.200462	newly 5 weeks spec	1875.	5403.49	{4982., 5657.}	188.186	0.459225	newly 5 weeks cumul	15610.	28870.7	{27164., 30799.}	84.9502	0.266662	newly 51 weeks cumul 19660. 58164.5 {52723., 61411.} 195.852 0.47031
3	newly 3 weeks spec	2615.	6618.93	{6132., 6986.}	153.114	0.402783	newly 3 weeks cumul	10083.	19148.5	{17560., 20221.}	89.9091	0.277965	newly 5 weeks spec	1275.	5933.64	{5544., 6257.}	365.384	0.667367	newly 5 weeks cumul	13206.	31491.4	{29161., 33123.}	138.463	0.376953	newly 54 weeks cumul 16122. 62272.3 {56721., 66848.} 286.256 0.586061
4	newly 3 weeks spec	3002.	8372.35	{8190., 8508.}	178.892	0.445271	newly 3 weeks cumul	14624.	28559.	{28110., 28901.}	95.2888	0.290578	newly 5 weeks spec	1149.	5407.17	{5233., 5561.}	370.598	0.672389	newly 5 weeks cumul	17569.	40811.8	{40301., 41047.}	132.294	0.365929	newly 58 weeks cumul 18847. 57550.2 {56498., 58164.} 205.355 0.484632
5	newly 3 weeks spec	2761.	6469.47	{6124., 6868.}	134.316	0.369388	newly 3 weeks cumul	10134.	19034.5	{18126., 20148.}	87.8281	0.27341	newly 5 weeks spec	1641.	5603.85	{5317., 6214.}	241.49	0.53275	newly 5 weeks cumul	14065.	30789.4	{29317., 32710.}	118.908	0.339882	newly 54 weeks cumul 17712. 57126.3 {54320., 65975.} 222.529 0.507589
6	newly 3 weeks spec	2631.	6286.3	{5683., 6719.}	138.932	0.377473	newly 3 weeks cumul	10483.	18765.5	{17023., 20348.}	79.0086	0.251978	newly 5 weeks spec	1399.	5385.69	{4931., 5710.}	284.967	0.584666	newly 5 weeks cumul	13754.	30090.4	{27395., 32114.}	118.775	0.339223	newly 52 weeks cumul 16963. 57460. {52307., 61362.} 238.737 0.529057
7	newly 3 weeks spec	3036.	9626.01	{9388., 9836.}	217.062	0.501082	newly 3 weeks cumul	13981.	29014.3	{28338., 29749.}	107.526	0.316993	newly 5 weeks spec	924.	7340.31	{7188., 7481.}	694.406	0.899984	newly 5 weeks cumul	16613.	44980.5	{44210., 45778.}	170.755	0.432534	newly 57 weeks cumul 17582. 72208.8 {71265., 72965.} 310.697 0.613506
8	newly 3 weeks spec	2036.	6673.84	{6480., 6917.}	227.792	0.515466	newly 3 weeks cumul	8038.	19417.	{18830., 20097.}	141.564	0.382902	newly 5 weeks spec	1057.	5948.11	{5759., 6133.}	462.735	0.750175	newly 5 weeks cumul	10545.	31777.2	{31012., 32724.}	201.348	0.478966	newly 51 weeks cumul 12212. 63042.2 {60174., 64714.} 416.232 0.712682
9	newly 3 weeks spec	3348.	8476.06	{8193., 8702.}	153.168	0.403286	newly 3 weeks cumul	14156.	27469.	{26302., 28287.}	94.0447	0.287744	newly 5 weeks spec	1368.	6027.09	{5819., 6219.}	340.577	0.643891	newly 5 weeks cumul	17721.	40794.5	{39060., 41792.}	130.204	0.361996	newly 56 weeks cumul 20118. 62274.7 {59433., 63974.} 209.547 0.490608
10	newly 3 weeks spec	2716.	5047.33	{4729., 5254.}	85.8369	0.268815	newly 3 weeks cumul	9804.	15101.6	{14238., 15686.}	54.035	0.187369	newly 5 weeks spec	1557.	4470.2	{4186., 4657.}	187.103	0.457674	newly 5 weeks cumul	13469.	24416.4	{22892., 25303.}	81.2786	0.258102	newly 50 weeks cumul 16876. 50680.4 {46722., 53266.} 200.311 0.477121
11	newly 3 weeks spec	3997.	7316.47	{7208., 7438.}	83.049	0.262534	newly 3 weeks cumul	15533.	21055.7	{20876., 21233.}	35.5544	0.132104	newly 5 weeks spec	1963.	6417.24	{6287., 6542.}	226.91	0.514378	newly 5 weeks cumul	20137.	34492.3	{34243., 34732.}	71.2883	0.23372	newly 54 weeks cumul 22930. 64055.3 {63747., 64374.} 179.351 0.446148
12	newly 3 weeks spec	3071.	6011.57	{5753., 6221.}	95.7528	0.291482	newly 3 weeks cumul	10922.	17917.8	{17279., 18490.}	64.0525	0.21479	newly 5 weeks spec	2036.	5275.84	{5073., 5448.}	159.128	0.413285	newly 5 weeks cumul	15499.	28919.7	{27993., 29780.}	86.5907	0.270709	newly 50 weeks cumul 21968. 57076.3 {55145., 58981.} 159.816 0.414433
13	newly 3 weeks spec	3153.	7790.73	{7415., 8127.}	147.089	0.392508	newly 3 weeks cumul	12554.	23715.7	{22753., 24878.}	88.9097	0.275931	newly 5 weeks spec	1478.	5912.01	{5606., 6119.}	300.001	0.601578	newly 5 weeks cumul	16279.	36580.6	{35134., 38097.}	124.71	0.351326	newly 55 weeks cumul 18456. 57150.6 {54665., 58635.} 209.658 0.490277
14	newly 3 weeks spec	2684.	7491.09	{7326., 7656.}	179.102	0.445645	newly 3 weeks cumul	12513.	25159.8	{24702., 25493.}	101.069	0.303237	newly 5 weeks spec	929.	5148.82	{4958., 5597.}	454.233	0.743267	newly 5 weeks cumul	15065.	36611.2	{36096., 37093.}	143.022	0.38556	newly 57 weeks cumul 16544. 54917.2 {53619., 59028.} 231.946 0.52079
15	newly 3 weeks spec	3944.	7903.98	{7789., 8029.}	100.405	0.301877	newly 3 weeks cumul	16141.	23455.1	{23232., 23679.}	45.314	0.162295	newly 5 weeks spec	1651.	6474.32	{6371., 6588.}	292.145	0.593409	newly 5 weeks cumul	20293.	37251.7	{36968., 37552.}	83.5691	0.263791	newly 54 weeks cumul 22727. 64128.4 {63795., 64410.} 182.168 0.450505
16	newly 3 weeks spec	2270.	4714.04	{4469., 4928.}	107.667	0.31704	newly 3 weeks cumul	7578.	13394.5	{12694., 14031.}	76.7546	0.247075	newly 5 weeks spec	1786.	4649.8	{4415., 4892.}	160.347	0.415227	newly 5 weeks cumul	11349.	22795.6	{21636., 23715.}	100.86	0.302621	newly 50 weeks cumul 16325. 55993.2 {53084., 58700.} 242.99 0.534934
17	newly 3 weeks spec	4099.	8900.05	{8390., 9282.}	117.127	0.336417	newly 3 weeks cumul	15754.	27552.2	{25415., 28852.}	74.8903	0.242296	newly 5 weeks spec	1851.	6687.77	{6510., 6882.}	261.306	0.557474	newly 5 weeks cumul	20371.	42131.2	{39834., 43653.}	106.82	0.315283	newly 56 weeks cumul 22951. 67362.5 {66888., 68896.} 193.506 0.467108
18	newly 3 weeks spec	3126.	10712.2	{10591., 10832.}	242.68	0.534857	newly 3 weeks cumul	17638.	32333.1	{32138., 32565.}	83.3151	0.263178	newly 5 weeks spec	665.	7850.06	{7727., 7964.}	1080.46	1.07201	newly 5 weeks cumul	19872.	49635.	{49384., 49957.}	149.774	0.39753	newly 60 weeks cumul 20225. 76776.4 {76504., 77075.} 279.611 0.57933
19	newly 3 weeks spec	3015.	6184.9	{6057., 6339.}	105.138	0.311904	newly 3 weeks cumul	11397.	18179.1	{17921., 18591.}	59.5075	0.202615	newly 5 weeks spec	1523.	5456.55	{5360., 5576.}	258.276	0.554153	newly 5 weeks cumul	15147.	29537.4	{29114., 30072.}	95.005	0.289956	newly 51 weeks cumul 18357. 59090.2 {58376., 59514.} 221.895 0.507681
20	newly 3 weeks spec	3891.	11460.7	{11307., 11623.}	194.543	0.469122	newly 3 weeks cumul	17885.	33763.6	{33359., 34073.}	88.7817	0.275945	newly 5 weeks spec	1246.	8528.51	{8366., 8701.}	584.471	0.835303	newly 5 weeks cumul	21430.	52483.9	{52005., 53012.}	144.909	0.388993	newly 60 weeks cumul 22520. 81543. {80978., 82269.} 262.092 0.558807
21	newly 3 weeks spec	3638.	10371.2	{9703., 10730.}	185.081	0.454569	newly 3 weeks cumul	16322.	33179.4	{31148., 34083.}	103.28	0.307867	newly 5 weeks spec	1041.	7003.45	{6650., 7364.}	572.762	0.825891	newly 5 weeks cumul	19258.	48920.7	{46459., 50238.}	154.028	0.404497	newly 60 weeks cumul 20284. 70717.4 {65995., 74001.} 248.636 0.540703
22	newly 3 weeks spec	3362.	5207.76	{5022., 5367.}	54.9007	0.189875	newly 3 weeks cumul	10760.	15369.	{14732., 15908.}	42.8341	0.154617	newly 5 weeks spec	2166.	4706.09	{4548., 4885.}	117.271	0.336802	newly 5 weeks cumul	15662.	25101.8	{24283., 25878.}	60.2717	0.204713	newly 50 weeks cumul 22682. 53803.2 {50043., 57548.} 137.207 0.374587
23	newly 3 weeks spec	3951.	8598.56	{8237., 8926.}	117.63	0.33749	newly 3 weeks cumul	16287.	25116.9	{24301., 25924.}	54.2142	0.18797	newly 5 weeks spec	1653.	7031.41	{6548., 7441.}	325.373	0.628185	newly 5 weeks cumul	20536.	40152.1	{38541., 41551.}	95.5203	0.290985	newly 55 weeks cumul 22473. 67538.8 {61409., 71828.} 200.533 0.476816
24	newly 3 weeks spec	2779.	10228.2	{9892., 10493.}	268.054	0.565746	newly 3 weeks cumul	13850.	33322.5	{32848., 33938.}	140.596	0.381221	newly 5 weeks spec	702.	6885.69	{6642., 7230.}	880.868	0.990431	newly 5 weeks cumul	16098.	48779.8	{48154., 49777.}	203.018	0.481308	newly 59 weeks cumul 16529. 70858.3 {70209., 73394.} 328.691 0.631278
25	newly 3 weeks spec	3462.	5383.64	{5194., 5549.}	55.5066	0.191633	newly 3 weeks cumul	12350.	15314.4	{14868., 15744.}	24.0036	0.0933439	newly 5 weeks spec	2037.	5202.25	{5064., 5352.}	155.388	0.407119	newly 5 weeks cumul	17226.	25905.6	{25315., 26533.}	50.3867	0.177141	newly 50 weeks cumul 22743. 61201.2 {60473., 61794.} 169.099 0.429899
26	newly 3 weeks spec	3048.	7069.95	{6654., 7410.}	131.954	0.365039	newly 3 weeks cumul	12655.	21061.6	{19942., 21994.}	66.4292	0.220915	newly 5 weeks spec	1843.	5999.96	{5668., 6286.}	225.554	0.512265	newly 5 weeks cumul	16877.	33693.6	{32022., 35108.}	99.6418	0.299949	newly 52 weeks cumul 20285. 63007.3 {60285., 65225.} 210.61 0.491965
27	newly 3 weeks spec	3519.	5482.09	{5346., 5597.}	55.7855	0.192458	newly 3 weeks cumul	12256.	15763.6	{15439., 16065.}	28.6191	0.109246	newly 5 weeks spec	1833.	5196.08	{5074., 5296.}	183.474	0.452449	newly 5 weeks cumul	16564.	26397.8	{26008., 26779.}	59.3683	0.202365	newly 50 weeks cumul 21086. 60535.9 {59953., 61225.} 187.091 0.458007
28	newly 3 weeks spec	2682.	5427.51	{5045., 5742.}	102.368	0.305613	newly 3 weeks cumul	8965.	15522.4	{14351., 16402.}	73.1446	0.237906	newly 5 weeks spec	1792.	5145.84	{4797., 5452.}	187.156	0.457621	newly 5 weeks cumul	12944.	26054.	{24242., 27454.}	101.282	0.303346	newly 51 weeks cumul 19275. 58104.2 {54084., 62052.} 201.449 0.478667
29	newly 3 weeks spec	2188.	6040.81	{5635., 6549.}	176.088	0.440354	newly 3 weeks cumul	8468.	16786.	{15700., 18198.}	98.2284	0.296497	newly 5 weeks spec	1093.	5882.61	{5475., 6305.}	438.208	0.73035	newly 5 weeks cumul	11210.	28774.3	{27041., 31103.}	156.684	0.408791	newly 53 weeks cumul 14641. 63338.2 {60071., 68239.} 332.609 0.635574
30	newly 3 weeks spec	3476.	9386.49	{9129., 9665.}	170.037	0.431335	newly 3 weeks cumul	15376.	29767.3	{28905., 30565.}	93.5959	0.286793	newly 5 weeks spec	1413.	6439.89	{6301., 6586.}	355.76	0.658662	newly 5 weeks cumul	19115.	44195.5	{43204., 45119.}	131.209	0.363944	newly 55 weeks cumul 20923. 63220.6 {62431., 63924.} 202.158 0.480216
31	newly 3 weeks spec	2766.	6619.55	{6348., 6982.}	139.319	0.378674	newly 3 weeks cumul	10733.	19012.	{18242., 19999.}	77.1361	0.24805	newly 5 weeks spec	1803.	6021.56	{5747., 6425.}	233.974	0.523325	newly 5 weeks cumul	14870.	31485.6	{30240., 33180.}	111.739	0.325538	newly 52 weeks cumul 18960. 64209. {62069., 68280.} 238.655 0.529445
32	newly 3 weeks spec	2860.	5500.63	{5149., 5795.}	92.3297	0.283664	newly 3 weeks cumul	9853.	16299.1	{15442., 17074.}	65.4227	0.218311	newly 5 weeks spec	1902.	4913.62	{4642., 5154.}	158.34	0.411864	newly 5 weeks cumul	14117.	26491.9	{25092., 27610.}	87.6596	0.273094	newly 50 weeks cumul 21462. 54748.4 {51520., 56995.} 155.094 0.406392
33	newly 3 weeks spec	1762.	11434.5	{11278., 11572.}	548.952	0.812189	newly 3 weeks cumul	12781.	32507.3	{32218., 32767.}	154.341	0.405407	newly 5 weeks spec	298.	8875.71	{8760., 8998.}	2878.43	1.47396	newly 5 weeks cumul	13856.	51813.2	{51492., 52162.}	273.941	0.572797	newly 60 weeks cumul 13996. 82985.4 {82604., 83376.} 492.922 0.772995
34	newly 3 weeks spec	2684.	8629.	{8480., 8773.}	221.498	0.507139	newly 3 weeks cumul	12876.	27969.2	{27606., 28293.}	117.22	0.33688	newly 5 weeks spec	799.	6125.41	{5939., 6290.}	666.635	0.884495	newly 5 weeks cumul	15119.	41481.	{40894., 41852.}	174.363	0.438311	newly 56 weeks cumul 15839. 63386.8 {62314., 64046.} 300.195 0.602249
35	newly 3 weeks spec	685.	11199.9	{11066., 11355.}	1535.02	1.2135	newly 3 weeks cumul	10337.	29791.9	{29541., 30002.}	188.206	0.459695	newly 5 weeks spec	47.	9504.44	{9359., 9653.}	20122.2	2.3058	newly 5 weeks cumul	10552.	50069.5	{49775., 50337.}	374.503	0.676235	newly 60 weeks cumul 10552. 85294.5 {85056., 85498.} 708.325 0.907585
36	newly 3 weeks spec	2872.	10630.2	{10479., 10782.}	270.132	0.568331	newly 3 weeks cumul	12863.	30675.9	{30344., 30969.}	138.481	0.377443	newly 5 weeks spec	910.	8228.29	{8086., 8380.}	804.208	0.956229	newly 5 weeks cumul	15281.	48582.8	{48270., 48872.}	217.93	0.502326	newly 60 weeks cumul 16833. 78153.3 {77769., 78498.} 364.286 0.666783
37	newly 3 weeks spec	3362.	7173.62	{7023., 7299.}	113.374	0.32909	newly 3 weeks cumul	13277.	21677.4	{21290., 22062.}	63.2702	0.212866	newly 5 weeks spec	1507.	5861.59	{5726., 5992.}	288.958	0.589836	newly 5 weeks cumul	17055.	34168.9	{33582., 34624.}	100.346	0.301753	newly 53 weeks cumul 19554. 59979.5 {59349., 60632.} 206.738 0.486749
38	newly 3 weeks spec	2972.	10930.7	{10782., 11081.}	267.789	0.565574	newly 3 weeks cumul	16469.	32335.5	{32033., 32646.}	96.3416	0.293	newly 5 weeks spec	612.	8180.54	{8014., 8338.}	1236.69	1.12599	newly 5 weeks cumul	18339.	50263.9	{49959., 50587.}	174.082	0.437875	newly 60 weeks cumul 18646. 78654.7 {78008., 79131.} 321.831 0.625133
39	newly 3 weeks spec	2387.	8886.77	{8601., 9108.}	272.299	0.570787	newly 3 weeks cumul	10499.	28412.9	{27788., 29051.}	170.625	0.432308	newly 5 weeks spec	937.	6350.11	{5997., 6580.}	577.707	0.830761	newly 5 weeks cumul	12897.	42437.	{41313., 43241.}	229.045	0.517181	newly 57 weeks cumul 14107. 64339.5 {60368., 66256.} 356.082 0.65873
40	newly 3 weeks spec	2220.	9988.43	{9848., 10147.}	349.929	0.653121	newly 3 weeks cumul	13427.	32801.9	{32557., 33022.}	144.298	0.387913	newly 5 weeks spec	616.	6727.46	{6623., 6855.}	992.12	1.03823	newly 5 weeks cumul	15217.	47889.8	{47592., 48163.}	214.712	0.497908	newly 59 weeks cumul 15737. 69838.4 {69490., 70271.} 343.785 0.647168
41	newly 3 weeks spec	3803.	10533.	{10387., 10675.}	176.966	0.442399	newly 3 weeks cumul	18778.	32675.5	{32499., 32872.}	74.0092	0.240567	newly 5 weeks spec	894.	7527.96	{7400., 7631.}	742.054	0.925307	newly 5 weeks cumul	21558.	49324.	{49049., 49561.}	128.797	0.359447	newly 60 weeks cumul 22565. 74754.6 {74553., 74933.} 231.286 0.520201
42	newly 3 weeks spec	3308.	9566.21	{9378., 9764.}	189.184	0.461111	newly 3 weeks cumul	14867.	30618.6	{30144., 31113.}	105.95	0.31371	newly 5 weeks spec	1090.	6702.02	{6505., 6857.}	514.864	0.788699	newly 5 weeks cumul	17792.	45503.9	{45096., 46118.}	155.755	0.407796	newly 57 weeks cumul 18900. 68416.6 {67639., 69352.} 261.993 0.558674
43	newly 3 weeks spec	4665.	10579.8	{9864., 10813.}	126.79	0.355428	newly 3 weeks cumul	19701.	32371.3	{29989., 32938.}	64.3131	0.215488	newly 5 weeks spec	1372.	7675.04	{7275., 7859.}	459.405	0.747588	newly 5 weeks cumul	23754.	49321.7	{45796., 50033.}	107.635	0.317139	newly 60 weeks cumul 25089. 75617.9 {71317., 76598.} 201.399 0.479041
44	newly 3 weeks spec	3246.	10476.1	{10329., 10618.}	222.737	0.508825	newly 3 weeks cumul	17555.	33446.	{33191., 33721.}	90.521	0.279934	newly 5 weeks spec	782.	7257.05	{7115., 7375.}	828.012	0.967511	newly 5 weeks cumul	19996.	49605.6	{49286., 49940.}	148.078	0.394582	newly 59 weeks cumul 20762. 73517.3 {73134., 73883.} 254.096 0.549117
45	newly 3 weeks spec	3565.	6984.11	{6569., 7361.}	95.9077	0.291674	newly 3 weeks cumul	13069.	21000.	{19947., 22081.}	60.6854	0.205658	newly 5 weeks spec	1772.	5625.94	{5281., 6223.}	217.491	0.500982	newly 5 weeks cumul	17480.	33027.7	{31473., 34752.}	88.9457	0.276012	newly 55 weeks cumul 20564. 55601. {51377., 64746.} 170.38 0.430574
46	newly 3 weeks spec	4749.	9687.59	{9519., 9847.}	103.992	0.309581	newly 3 weeks cumul	18136.	30025.2	{29503., 30471.}	65.5558	0.218916	newly 5 weeks spec	1998.	7102.89	{6987., 7233.}	255.5	0.550792	newly 5 weeks cumul	23379.	45631.1	{45109., 46050.}	95.1799	0.290422	newly 56 weeks cumul 26044. 70976.3 {70414., 71539.} 172.524 0.435395
47	newly 3 weeks spec	3376.	9192.7	{8938., 9419.}	172.296	0.434951	newly 3 weeks cumul	16086.	29279.1	{28412., 29887.}	82.0159	0.260025	newly 5 weeks spec	1272.	6565.4	{6381., 6758.}	416.148	0.712642	newly 5 weeks cumul	19497.	43793.5	{42706., 44737.}	124.617	0.351381	newly 56 weeks cumul 20865. 67069.1 {65668., 68380.} 221.443 0.50706
48	newly 3 weeks spec	3671.	5584.52	{5485., 5690.}	52.1253	0.18216	newly 3 weeks cumul	14471.	15616.2	{15449., 15776.}	7.91362	0.0330603	newly 5 weeks spec	1686.	5521.85	{5396., 5638.}	227.512	0.515169	newly 5 weeks cumul	18539.	26804.5	{26584., 27010.}	44.5846	0.16011	newly 51 weeks cumul 21237. 64101.1 {63731., 64456.} 201.837 0.479768
49	newly 3 weeks spec	2539.	8861.39	{8329., 9166.}	249.011	0.542574	newly 3 weeks cumul	11374.	26422.5	{25298., 27228.}	132.306	0.365855	newly 5 weeks spec	806.	6970.62	{6405., 7298.}	764.841	0.9363	newly 5 weeks cumul	13685.	41477.9	{39044., 42733.}	203.09	0.481329	newly 57 weeks cumul 14608. 68429.7 {61816., 72122.} 368.44 0.669674
50	newly 3 weeks spec	2519.	8805.43	{8403., 9602.}	249.561	0.542988	newly 3 weeks cumul	12653.	28528.9	{27551., 30934.}	125.472	0.352648	newly 5 weeks spec	744.	6051.08	{5652., 6668.}	713.317	0.909553	newly 5 weeks cumul	14778.	42039.7	{40540., 45677.}	184.475	0.453563	newly 58 weeks cumul 15627. 61799.2 {58843., 68075.} 295.464 0.596452
